# The Role of Nile Tilapia (*Oreochromis niloticus*) in the Life Cycle of *Toxocara* spp.

**DOI:** 10.3389/fvets.2021.685911

**Published:** 2021-06-17

**Authors:** Everton André de Oliveira, Yslla Fernanda Fitz Balo Merigueti, Isabella Braghin Ferreira, Isabele Santos Garcia, Alini Soriano Pereira, Rosemeire de Souza Santos, Louise Bach Kmetiuk, Andrea Pires dos Santos, Alexander Welker Biondo, Rogerio Giuffrida, Vamilton Alvares Santarém

**Affiliations:** ^1^Graduate College in Animal Science, São Paulo Western University, Presidente Prudente, Brazil; ^2^Laboratory of Veterinary Parasitology, Veterinary Teaching Hospital, São Paulo Western University, Presidente Prudente, Brazil; ^3^Laboratory of Pisciculture, Zootechny Teaching Aquaculture, São Paulo Western University, Presidente Prudente, Brazil; ^4^Graduate College of Molecular Biology, Federal University of Paraná, Curitiba, Brazil; ^5^Department of Comparative Pathobiology, College of Veterinary Medicine, Purdue University, West Lafayette, IN, United States; ^6^Department of Veterinary Medicine, Federal University of Paraná, Curitiba, Brazil

**Keywords:** environmental contamination, fish, toxocariasis, transmission, zoonosis

## Abstract

The present study aimed to experimentally assess Nile tilapia as potential paratenic host of *Toxocara* spp. A total of 15 Nile tilapia (*Oreochromis niloticus*) were fed with 300 embryonated *Toxocara canis* eggs by oral gavage, while five others of the control group received distilled water. The fish were individually analyzed at 16, 24, 48, 72, and 240 h after inoculation. Water contamination was assessed, and tissue migration by liver, gastrointestinal tract (GIT), eyes, and central nervous system. A murine model was used as the paratenic host for egg infectivity assessment. Eggs and larvae were found in plastic tank water and fish GIT, ranging from 23 to 86% per fish. Eggs and larvae were recovered from the tank water (76.3%) and fish GIT (23.7%). The counting of eggs and larvae observed was negatively correlated with number of eggs and larvae in the water tank (rho = −0.698, *p* = 0.003). Shedding of embryonated eggs was first detected at 16 and up to 240 h, with significant egg and larvae yield decrease on water-shedding (*p* = 0.001) and in the GIT (*p* = 0.007). Although no *T. canis* larva was recovered in fish tissues, egg infectivity after fish GIT transit was experimentally confirmed by mice assessment. In conclusion, despite shedding viable embryonated eggs through the gastrointestinal tract, tilapias may not play a role as a suitable paratenic hosts for *Toxocara* spp., posing low risk of zoonotic transmission by fish meat consumption.

## Introduction

The consumption of raw or inadequately cooked fish has been increasingly popular throughout the world (1, 2). Several parasitic zoonotic agents may be related to such fish and seafood consumption, including *Anisakis* spp. (3, 4), *Gnathostoma* spp. (5), and *Toxoplasma gondii* (6, 7). In addition, contamination of fish-based dishes during handling may also contribute to spreading zoonotic diseases (8).

Toxocarosis has been considered one of the most prevalent parasitic zoonoses, particularly in vulnerable populations (9–11). Despite the widely used term “toxocariasis,” toxocarosis has been the standardized nomenclature of this animal parasitic disease (10, 11). Toxocarosis is among the six most important neglected parasitic infections in the USA, along with Chagas disease, cyclosporiasis, cysticercosis, toxoplasmosis, and trichomoniasis, due to its high prevalence, chronic and disabling characteristics, and a strong link with poverty (12).

Although most human infections have been asymptomatic (13), systemic larval migration through organs may cause liver damage (14), respiratory symptoms and other disorders such as asthma (15, 16). Ocular toxocarosis may lead to vision impairment, strabismus, leukocoria and retinal granulomatous lesion (17, 18). *Toxocara* larvae can cross the blood-brain barrier, invading the central nervous system (neurotoxocarosis), leading to meningitis, encephalitis, myelitis and cerebral vasculitis (19, 20).

Toxocarosis has been primarily associated with the ingestion of *Toxocara* spp. eggs from the soil (21). The definitive hosts of *Toxocara canis* and *Toxocara cati*, respectively, dogs and cats, play an important role in the oral–fecal transmission cycle, by excreting eggs directly into the anthropic environment, including recreational, public and urban green areas (22–24). As shown in a recent meta-analysis study, a fifth of public areas worldwide has been contaminated with *Toxocara* spp. eggs, indicating that soil may be a major source of toxocarosis and public health concern (25), which has been associated to presence of stray dogs with a higher number of positive fecal samples for intestinal nematode eggs (26). Prevalence of anti-*T. canis* antibodies has been extensively studied in dogs throughout the world, including 188/7,409 (2.54%) owned dogs of North America (27); 7/200 (3.5%) owned and sheltered dogs in Greece (28); 11/239 (4.6%) owned dogs of Belgium and Netherlands (29) and 157/296 (53.04%) dogs of Egypt (30). Meta-analysis studies have estimated a global 11.1% prevalence in dogs (31) and of 17.0% in cats (32), whereas the global human seroprevalence was estimated in 19.0% (33).

History of intaking raw meat of paratenic hosts such as sheep (34), rabbits (35), cattle (36), domestic pigs (37), chickens (38, 39), and ostriches (40), have also been considered a risk factor for toxocarosis (33, 41, 42). Humans are considered accidental hosts of *Toxocara* spp. and are most commonly infected by ingesting embryonated eggs from soil or larvae from paratenic host tissues (9, 21, 43). In this species, the larvae may migrate to the small intestine and other organs, but the parasite is unable to complete its cycle (37, 38, 44, 45).

The assessment of *T. canis* in livestock animals helps prevent disease transmission (46), however, the role of fish in the epizootiological chain of *Toxocara* spp. remains unclear. In addition, companion animals such as dogs and cats are often maintained close to lakes, rivers, and ponds, including fish farms, resulting in water contamination and exposure of the fish to dog and cat feces (47). Consequently, infected fish later be consumed by dogs and human beings (48).

The Nile tilapia (*Oreochromis niloticus*) has been considered one of the most common freshwater-bred fish species worldwide (49, 50), because of their fast growth, hardiness, omnivore diet, resistance to low oxygen concentrations, easy farm management, and pleasant flavor with fewer bones (51). *Toxocara* eggs in open water from infected dogs and cats may also embryonate without fish presence and may develop into infective stages. Moreover, the fish gastrointestinal tract is mostly removed before human consumption, and when left it may be killed or inactivated by cooking or microwave, as previously shown (52). However, the role of raw fish meat as foodborne toxocarosis source remains to be fully established. Accordingly, this study aimed to experimentally assess Nile tilapia as potential paratenic host of *Toxocara* spp.

## Method

### Fish Selection and Maintenance

This study has been approved by the Ethics Committee of Animal Use of the São Paulo Western University (UNOESTE) (Protocol Number 4,299). Nile tilapias ranging from 7 to 10 cm in size, weighing between 20 and 31 g, and ~3 months old were randomly obtained in the fish farming section of the Zootechnical Center at the São Paulo Western University (UNOESTE). The fish was first transferred to a depuration tank with a water recirculation system and air compressor aeration for 7 days before the experiment, as previously recommended (50).

During the adjustment period, a fecal examination of each tilapia using flotation and centrifugal sedimentation (53) was performed to ensure the absence of coccidia and helminths.

After inoculation with *T. canis* eggs, each fish was individually housed in a 5-liter polyethylene tank throughout the post-inoculation period. Fish tanks were maintained in a controlled environment with 12-h light-dark cycles at 25 ± 5°C, constant water tank oxygenation by an air compressor, and fed twice a day until apparent satiation with commercially available fish food (Acqua 32 Matsuda®, São Paulo, Brazil).

### Experimental Design

Egg shedding into tank water and fish larvae migration were assessed at 16, 24, 48, 72, and 240 h post-inoculation. The inoculated group (IG) consisted of 15 fish inoculated with 300 *T. canis* embryonated eggs, and the control group (CG) consisted of five fish inoculated with distilled water (Table 1). Three inoculated and one control fish were euthanized and examined for larvae migration in each post-inoculation time.

**Table 1:** Assessment of *T. canis* eggs of the aquatic environment (^*^) and larvae in fish tissues (+), after Nile tilapia experimental inoculation [inoculated group (IG), *n* = 15] with embryonated eggs.

**Hours post-inoculation**					
**Group (number of evaluated fish)**	**16**	**24**	**48**	**72**	**240**
IG (3)	^*^+	NE	NE	NE	NE
IG (3)	NE	^*^+	NE	NE	NE
IG (3)	NE	^*^	^*^+	NE	NE
IG (3)	NE	^*^	^*^	^*^+	NE
IG (3)	NE	^*^	^*^	^*^	^*^+
CG (5)	^*^+	^*^+	^*^+	^*^+	^*^+

*One uninfected fish was evaluated at each time period [control group (CG), n = 5]*.

*NE, Not evaluated*.

A period of 16 h was set as the first fish assessment due to the minimal amount of time for food to pass into the intestine in Nile tilapia, as previously observed (54). Only one subgroup of (*n* = 3) fish was assessed at 16 h to avoid sampling stress on transfer to another tank in such a short interval.

### Recovery of *Toxocara canis* Eggs

*Toxocara canis* eggs were recovered according to a previously described protocol (55), with minor modifications. In short, adult *T. canis* females were recovered from feces shed by naturally infected puppies. Adult female parasites were washed with saline solution and hysterectomized. Eggs were incubated in 2% formalin solution for at least 30 days at 25 ± 2°C.

After incubation and confirmed embryonation, the eggs were washed with saline solution and centrifuged at 697 g for 3 min, the embryonated eggs were then placed on histological slides, and 300 units counted, as previously established (56). Eggs were transferred to plastic tubes containing 20 μL of distilled water, later used for fish inoculation.

### Fish Inoculation

Fish were sedated by immersion in an anesthetic solution of 50 mg of benzocaine diluted in ethanol until loss of equilibrium, as previously established (57). A total of 300 *T. canis* embryonated eggs were administered by oral gavage with a needle designed for mice (55). An additional 20 μL of distilled water was then administered to ensure successful egg ingestion. After inoculation, fish were individually monitored until equilibrium and external stimuli recovery and stabilization and then housed into a 5-L polyethylene tank throughout the post-inoculation period. Fish in the CG were orally given the same volume of 40 μL of distilled water.

### Egg Shedding Assessment

Tank water was filtered through 212- and 38-μm metal sieves to collect *T. canis* eggs. The filtered material was collected using a plastic pipette, transferred to a conical bottom tube (15 mL), and centrifuged at 697 g for 3 min to concentrate sediments. After centrifugation, the supernatant was discarded, and sediment was observed under an optical microscope at 100× and 400× magnifications to quantify recovered eggs.

After each egg shedding observation, the fish were individually transferred to new tanks with clean water, while old tanks were flushed with abundant water and filtered to obtain eggs potentially adhered to tank plastic walls.

### Assessment of *T. canis* Larvae in Fish Tissues

As already described, three inoculated and one control fish were euthanized and examined at 16, 24, 48, 72, and 240 post-inoculation hours to assess larval tissue migration (Table 1). Fish were immersed in an anesthetic solution of 50 mg of benzocaine diluted in ethanol, following previously described protocol (57). After observing no opercular movement and permanent stasis at the plastic tank bottom, fish were euthanized by spinal cord section (58).

Fish were dissected using forceps and scalpel blade; liver, stomach, intestines, gills, eyes, central nervous system, and lateral portion of the fish's musculature were extracted. Each organ was individually grounded in Petri dishes and subjected to acid digestion with 5 g pepsin and 10 mL HCl 37% in distilled water for 6 h at 37°C under agitation, as recommended (59). After digestion, tissue material was filtered through a 300 μm sieve and centrifuged at 697 g for 3 min.

Egg and larvae assessment in the GIT included stomach and intestines. Larvae were assessed thoroughly observing the final material with a light microscope at 100× and 400× magnifications.

### Assessment of *T. canis* Egg Infectivity in Mice

Two 5–7 weeks old male Swiss mice (*Mus musculus*), weighing ~50 g were maintained in a controlled environment at the Experimental Laboratory at UNOESTE, with 12-h light-dark cycles at 22 ± 2°C, and provided with commercial food and water *ad libitum*.

Assessment of *T. canis* egg infectivity after passing throughout the fish GIT was based on bioassay, as previously described (35). One tilapia was inoculated with embryonated (*n* = 300) and one with unembryonated (*n* = 300) *T. canis* eggs, following the same procedure described previously, except that eggs were retrieved from the tank water after 48 post-inoculation.

Following retrieval, 50 eggs shed by the tilapia inoculated with embryonated eggs were used for one mice inoculation. Recovered unembryonated eggs were used to inoculate the other mice but first maintained in 2% formalin solution in a temperature-controlled environment (27 ± 3°C) for embryonation and larval development. Then, the material was washed three times with saline solution by centrifugation at 679 g for 3 min, and 50 eggs were counted for inoculation.

The inoculation was achieved by oral gavage with 100 μL of buffered saline solution containing 50 eggs in each mouse, following a protocol previously described (60). The two mice were euthanized in a CO_2_ chamber 48 h after inoculation and necropsied for liver extraction. Larvae recovery was achieved using 1 g of the liver samples subjected to the previously described Baermann technique (41).

### Statistical Analysis

The dispersion was assessed, assuming that the concentration of eggs and larvae in the fish and water tank could be described by a Poisson distribution or negative binomial distribution (61, 62). Thus, a generalized linear model was proposed, in which the dependent variable was the count of eggs and larvae and the time in hours was the independent variable. The counts of eggs and larvae were super-dispersed, so a negative binomial distribution was used to describe the data (63).

Spearman non-parametric correlation analysis was used to describe the relationship between the counts of eggs and larvae in fish and water tank, accumulated for all evaluated time points. A significance of *p* < 0.05 was used. The statistical analysis was performed using R (64).

## Results

The recovery rate of egg and larvae shedding and retained in fish GIT ranged from 23 to 86% (mean = 48.4%) per fish. Approximately three-quarters of eggs and larvae were recovered from the water tank (76.3%), and one-quarter of eggs and larvae from the GIT (23.7%). The shedding of embryonated eggs was first detected 16 h post-inoculation and was observed until the end of the experiment at 240 h.

**Figure 1:**
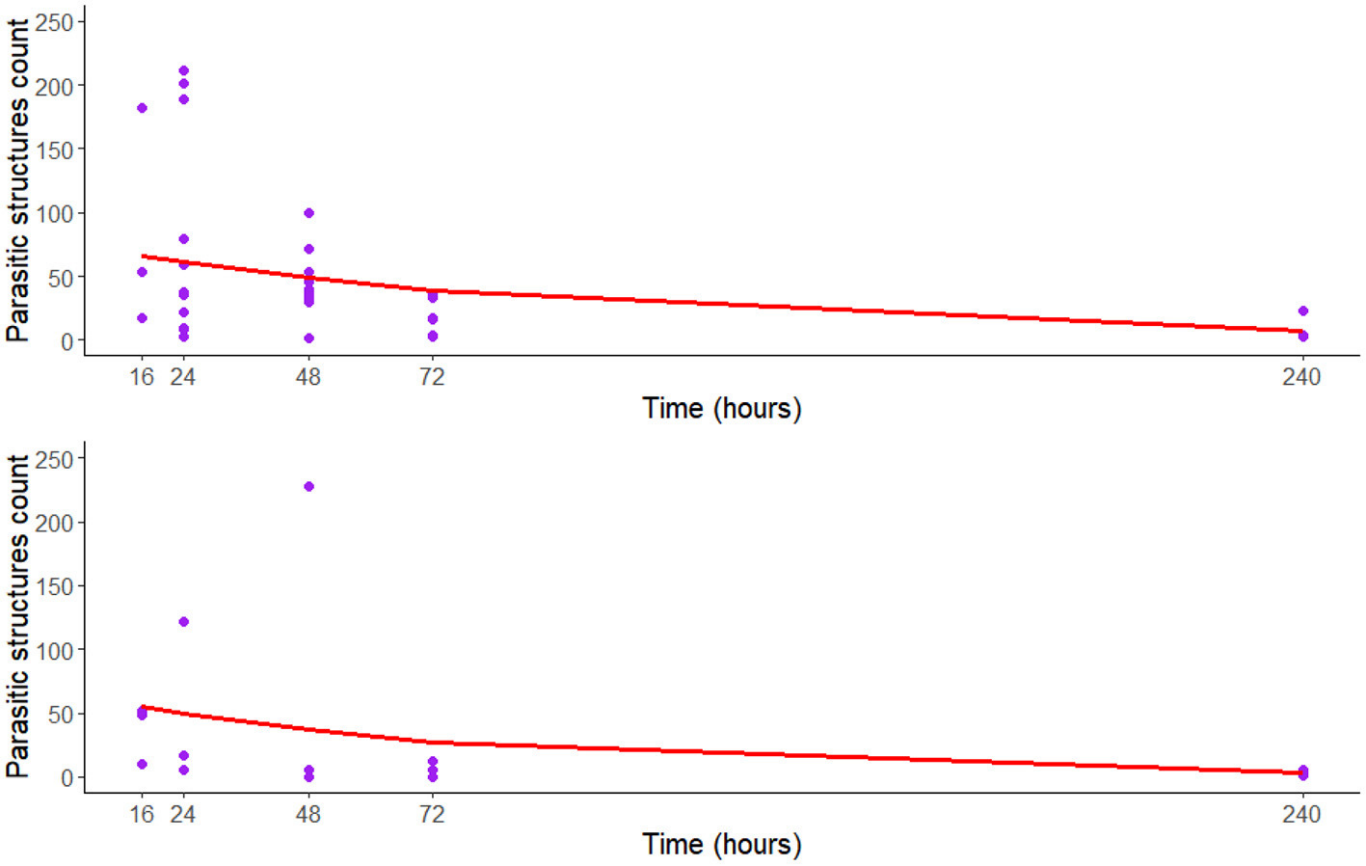
Regression curve of the negative binomial model to assessment *T. canis* eggs and larvae recovered of aquatic environment (upper) and in the gastrointestinal tracts (GIT) of Nile tilapia (lower) experimentally inoculated with *T. canis* embryonated eggs, overtime (*n* = 15).

The counting of eggs and larvae observed in the fish GIT was negatively correlated with the number of eggs and larvae identified in the water tank (rho = −0.698, *p* = 0.003). Regression models have shown a statistically significant decrease over time in egg shedding into the water tank (*p* = 0.001) and the presence of *T. canis* eggs in the fish GIT (*p* = 0.007; Table 2 and Figure 1).

**Table 2:** Negative binomial regression model to assessment *T. canis* eggs and larvae recovered of the aquatic environment and in the gastrointestinal tracts (GIT) of Nile tilapia experimentally inoculated with *T. canis* embryonated eggs, overtime.

**Parameter**	**Estimate**	**Standard error**	***Z* statistics**	***p-value***	**AIC**
***T. canis*** **of the aquatic environment**					
Intercept	4.342	0.244	17.74	<0.001	322.9
Hours post-inoculation	−0.010	0.003	−3.17	0.001	
***T. canis*** **of GIT of fish**					
Intercept	4.161	0.478	8.676	<0.001	194.76
Hours post-inoculation	−0.013	0.005	−2.697	0.007	

*p-value = Statistical significance of the regression coefficients associated with the Z-statistic; AIC, Akaike information criterion*.

Although some larvae were found in the water tank and fish GIT, no larvae were recovered from fish tissues. However, the bioassay has shown larvae in the digested liver of both mice, recovered at 48-h post-inoculation, with 5/50 (10.0%) and 9/50 (18.0%) larvae, for inoculation with unembryonated and embryonated eggs, respectively.

No fish died or presented any behavioral changes in either fish group. No other eggs and larvae were identified in the water, GIT, or in fish tissues. After abundantly washing the metallic meshes with water, no egg was retained in the sieves used to filter the plastic water tanks' material.

## Discussion

The presence of *T. canis* eggs into the water tanks herein has confirmed the capacity of egg shedding and dispersion into the aquatic environment by the Nile tilapia, as previously observed in other paratenic hosts and mechanical carriers experimentally infected with *T. canis* embryonated eggs, such as chickens (60), and cockroaches (65, 66). Not surprisingly, toxocarosis can be transmitted to human beings by ingestion of invertebrate hosts such as snails (67, 68) and earthworms (69), which may play a role as paratenic, mechanical, or biological hosts.

The egg recovery rate was 48.4% on average. Eggs were observed in tanks after 16 h post-inoculation, and fewer eggs were observed over time. The higher shedding of eggs occurred at 24 and 48 h post-inoculation, as previously observed in cockroaches inoculated with *T. canis* eggs (66). As expected, egg shedding was inversely proportional to the larvae presence in the tilapia GIT, consistent with observations in chicken (60) and cockroaches (65). The wide variation herein in egg and larval recovery per fish (23 to 86%) shows the absence of an egg dispersal pattern as observed in chickens (60).

In this study, we applied standard metallic sieves of different mesh sizes, which have been used to recover *Toxocara* spp. eggs from dog or cat fur (70, 71) and soil samples (72, 73). However, no egg was retained in the meshes employed for filtering the organic material from the plastic water tanks.

*Toxocara* spp. eggs, especially those of *T. canis*, tend to adhere to different materials, especially plastic (74). It is possible that the plastic water tanks may have favored egg adhesion and influenced egg recovery, despite the abundant washing during the filtering process.

As previously established, some nematodes may be transmitted by ingestion of raw/undercooked fish, after dogs/cats shed unembryonated eggs in water where they become embryonated, and larvae develop in the tissue of the intermediate fish host, as observed in *Gnathostoma* spp. (75). Despite the presence of eggs in the tank water and the GIT of the fish, no evidence of *T. canis* larvae migration into fish tissue was observed herein, as already observed in cockroaches and dog puppy tissues that were experimentally infected with feces of cockroaches containing embryonated eggs and larvae of *T. canis* (65). The authors hypothesize that the absence of migration may be due to the fish's poikilothermic characteristics, with GITs providing poor quality conditions for *T. canis* larvae hatching and tissue migration. On the other hand, homeothermic species such as dogs and cats have been confirmed as definitive hosts of *Toxocara* spp., and other homeothermic animals effectively play a role as intermediate hosts (76).

In this study, a few undamaged motile larvae were retrieved from tanks and the GITs of infected tilapia, which may be likely due to the rupture of eggs during sample processing, since eggshells mainly were observed near the larvae. Both embryonated and unembryonated *T. canis* eggs have the potential of infecting mice in experimental conditions after passing throughout the tilapia GIT, as observed in chicken (60). Although no larvae were found in Nile tilapia tissues herein, *T. canis* eggs and larvae were present in the tilapia GIT, and shedding eggs and larvae sustained infectivity for mice bioassay. Thus, Nile tilapia and other commercial fish may play a dispersion role of viable *T. canis* eggs.

Nile tilapia's ability to disperse *T. canis* eggs into the water tank should be considered in the epidemiological chain of toxocarosis. Non-dewormed dogs and cats infected by *Toxocara* spp. may live near fish breeding farms, and their feces can contaminate ponds and tanks used for fish farming (77). Dogs often enter the water to cool off, particularly on hot days (78), which may contaminate water resources and maintain the life cycle of parasites that have fish and other aquatic animals as intermediate or paratenic hosts (77). The presence of non-dewormed companion animals should be a public health concern in commercial fish farms. Restricting pet access to fish farms, feeding care, and pet regular anthelmintic treatment can reduce water resources contamination by parasite eggs (48, 79).

Despite *Toxocara* eggs in open water from infected dogs and cats may also embryonate without the presence of tilapia fish and develop into infective stages, the study herein has shown no evidence of larvae in the tilapia tissues. Thus, as tilapia act not as paratenic host, intake of raw fish meat may present no alimentary zoonotic infection risk. The fish, like other animals (e.g., dogs with coprophagy that eat cat feces) and human beings will just (partially or fully) pass developed eggs trough the gastrointestinal tract after ingestion, which may be removed from fish before human consumption. Although fish contamination with (embryonated) eggs may be possible with water contamination, as previously observed in irrigated vegetables (80), the most important conclusion from the present study has been that tilapia is not a suitable paratenic host for *Toxocara* spp.

One limitation of our study is the low number of animals included in the experimental design. Further studies should be conducted considering a larger number of animals and different dilution of embryonated eggs, to assess the sensibility of the fish to became infected. Nonetheless, to the authors knowledge, this is the first experimental study in which Nile tilapia has been tested, as previously experimentally performed in several other host species, infected with *T. canis* embryonated eggs to assess its role of infection carrier and potential risk of foodborne transmission.

Finally, as cross-contamination and human infection by parasitic agents may also occur during the handling and preparation of fish-based dishes (8), appropriate management practices, including training of fish handlers and workers in the production fish chain may minimize the impacts of fish-transmitted zoonoses.

The study herein has been the first experimental *T. canis* infection in Nile tilapia to assess its role as carrier. In conclusion, despite shedding viable embryonated eggs through the gastrointestinal tract, tilapias may not play a role as a suitable paratenic hosts for *Toxocara* spp., posing low risk of zoonotic transmission by fish meat consumption.

## Data Availability Statement

The original contributions presented in the study are included in the article/supplementary material, further inquiries can be directed to the corresponding author.

## Ethics Statement

This animal study was approved by the Ethics Committee of Animal Use of the São Paulo Western University—Brazil (protocol number 4299).

## Author Contributions

EO, YM, IF, IG, AP, RS, and VS performed experimental study and analysis. EO, RG, and VS wrote the first draft of the manuscript. EO, YM, IF, IG, AP, RS, LK, AS, AB, RG, and VS wrote sections of the manuscript. All authors contributed to data collection, data analysis, manuscript revision, read, and approved the submitted version.

## Conflict of Interest

The authors declare that the research was conducted in the absence of any commercial or financial relationships that could be construed as a potential conflict of interest.
